# Sodium–glucose cotransporter 2 inhibitor ameliorates podocyte hypertrophic stress by suppressing the mTOR/p70S6K/cyclin D1 signaling pathway in obesity-related nephropathy

**DOI:** 10.1038/s41598-026-49025-w

**Published:** 2026-04-20

**Authors:** Miho Suzuki, Akihiro Fukuda, Ryo Kurimoto, Akiko Kudo, Hirotaka Shibata

**Affiliations:** Department of Endocrinology, Metabolism, Rheumatology and Nephrology, Faculty of Medicine, Oita University, Yufu, 879-5593 Japan

**Keywords:** mTOR/p70S6K/cyclin D1 signaling pathway, Obesity-related nephropathy, Podocyte hypertrophic stress, SGLT2 inhibitor, Diseases, Nephrology, Physiology

## Abstract

Obesity-related nephropathy (ORN) is a risk factor for chronic kidney disease, end-stage kidney disease, and increased mortality. However, the underlying mechanism and treatment of ORN remain unknown. Compared to Zucker lean rats (control), Zucker fatty (ZF) rats, a nondiabetic obesity model, showed marked increases in body weight, urinary protein excretion, urinary sediment podocin (U-sed pod) mRNA levels, and glomerular/podocyte hypertrophy with age, implying that podocyte hypertrophic stress is linked to ORN development. Conversely, sodium–glucose cotransporter 2 inhibitor (SGLT2i) administration decreased body weight, urinary protein, U-sed pod mRNA excretion, and glomerular/podocyte hypertrophy. RNA sequencing of the renal cortex in ZF rats showed increased cyclin D1 expression and elevated protein levels of cyclin D1, IGF-1, and phospho S6 levels, indicating that IGF-1-mediated activation of the mTOR/p70S6K/cyclin D1 signaling pathway is involved in ORN. These changes were restored via SGLT2i treatment. Furthermore, the protective effects of SGLT2i on podocytes partially exceeded those of calorie restriction equivalent to the calorie loss from urinary glucose excretion. These results indicate that SGLT2i suppresses the IGF-1-mediated mTOR/p70S6K/cyclin D1 signaling pathway and reduces glomerular/podocyte hypertrophic stress. This represents a novel mechanism for the podocyte-protective effect of SGLT2i and underscores their potential as a therapeutic strategy for ORN.

**Supplementary Information:**

The online version contains supplementary material available at 10.1038/s41598-026-49025-w.

## Introduction

The prevalence of kidney diseases caused by lifestyle factors, particularly obesity-related nephropathy (ORN), has increased. Chronic kidney disease (CKD) was the 17th leading cause of death in 1990 and it emerged as the 12th leading cause in 2017^1^. In the United States, obesity is associated with 24%–33% of renal disease cases^2^. Furthermore, the incidence of obesity-related glomerulopathy in renal biopsies gradually increased from 0.2% in 1986–1990 to 2.0% in 1996–2000, showing a tenfold increase in 15 years^3^. In 2022, the number of obese adults was 880 million worldwide^4^, highlighting the critical public health challenge posed by obesity. Obesity is an independent risk factor for CKD development, and ORN frequently progresses to end-stage kidney disease^5,6^. The mechanisms underlying ORN must be understood.

Several pathophysiological mechanisms have been linked to ORN development, including glomerular hyperfiltration^7,8^, overactivation of the renin–angiotensin–aldosterone system (RAAS)^9–11^, hyperinsulinemia and insulin resistance^12,13^, elevated inflammatory cytokine levels^14,15^, ectopic lipid accumulation and lipotoxicity^16,17^, and oxidative stress^18,19^. However, these mechanisms are not completely understood. ORN’s hallmark pathological findings include glomerular enlargement and focal segmental glomerulosclerosis, with podocyte injury recognized as a major developmental factor^20,21^. Podocytes, an essential element of the glomerular filtration barrier, cover the surface of glomerular capillaries via foot processes and are required for glomerular function^22,23^. In ORN, podocyte foot process effacement and detachment from the basement membrane are observed as podocyte injury and depletion^21^. Reportedly, the accumulation of free fatty acids in podocytes causes insulin resistance and apoptosis^24^ and increased endoplasmic reticulum stress leads to podocyte loss^25^. Wiggins et al. proposed the podocyte depletion hypothesis—podocyte loss caused by persistent podocyte injury from various factors is the primary cause of glomerulosclerosis in many glomerular diseases, including obesity^26^. Furthermore, podocyte hypertrophic stress was detected in the early stages of a rat model and patient with type 2 diabetes before the onset of albuminuria, and its persistence led to nephropathy^27^. These findings imply that persistent podocyte injury due to podocyte hypertrophic stress may lead to ORN.

Although RAAS blockade and weight loss are effective in ORN, several patients continue to develop progressive nephropathy. At present, sodium–glucose cotransporter 2 inhibitors (SGLT2is) are available for treating CKD^28,29^. Although the protective effect of SGLT2i on podocytes in diabetic nephropathy has been reported in the recent years^30,31^, to our knowledge, no studies have reported whether podocyte injury is associated with ORN development and whether SGLT2i has a podocyte-protective effect. This study aimed to reveal the mechanism of podocyte injury-induced ORN progression and evaluate the podocyte-protective effects of SGLT2i.

## Results

### Progression of ORN due to podocyte hypertrophic stress

#### Time course

**Fig. 1:**
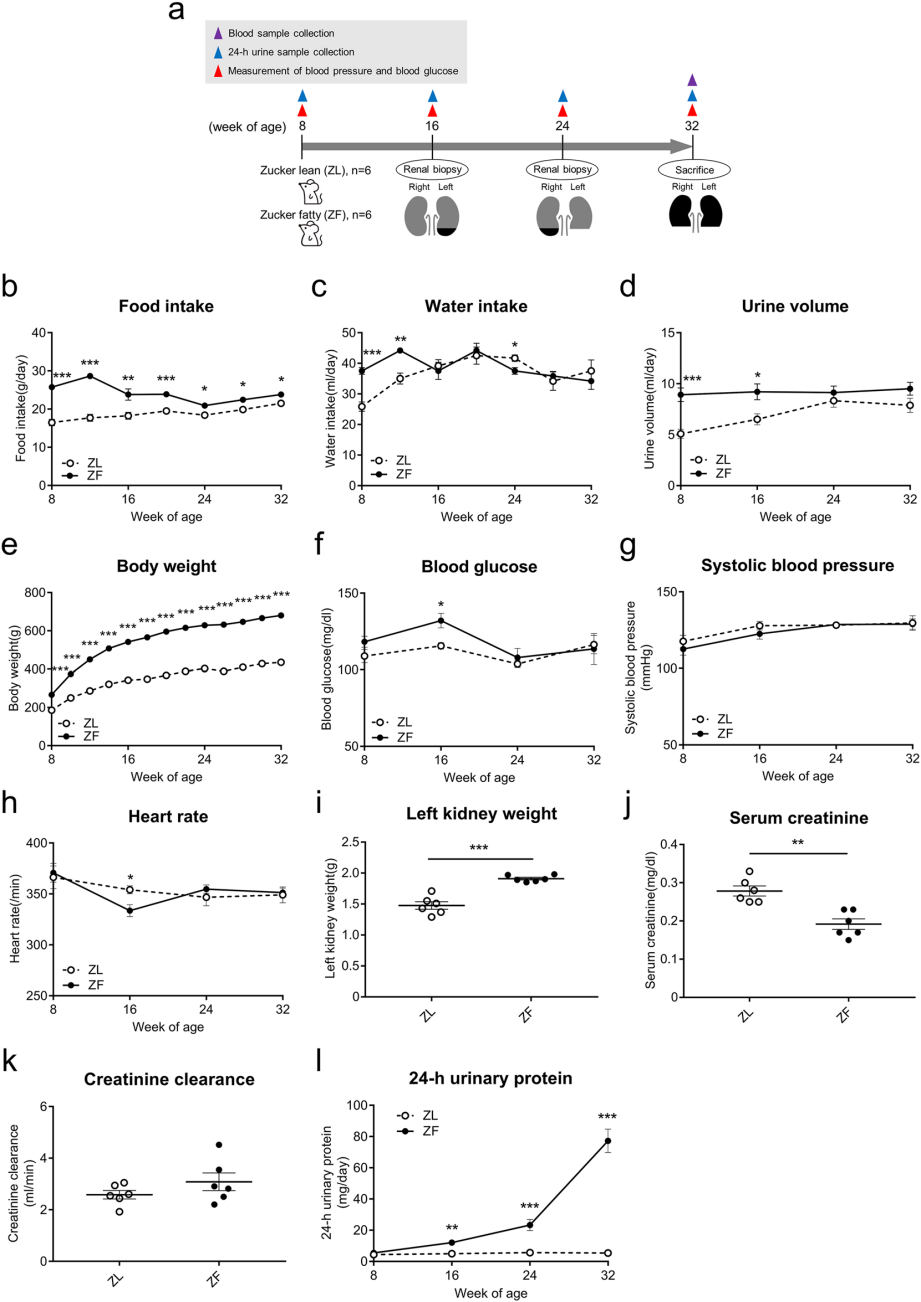
Time course of clinical parameters in Zucker lean (ZL) and Zucker fatty (ZF) rats. **a** Schematic representation of experimental protocols. **b** 24-h food intake. **c** 24-h water intake. **d** 24-h urine volume. **e** Body weight gain. **f** Blood glucose. **g** Systolic blood pressure. **h** Heart rate. **i** Left kidney weight at 32 weeks of age. **j** Serum creatinine. **k** Creatinine clearance calculated from urinary creatinine, serum creatinine, and urine volume at 32 weeks of age. **l** 24-h urinary protein. ZF rats gained weight and excreted more urinary protein over time. At 32 weeks of age, kidney weight increased, and creatinine clearance trended upward in ZF rats, implying that nephropathy and glomerular hyperfiltration may develop as obesity progresses. Data are presented as the mean ± SEM. Statistically significant differences are shown as: *P < 0.05, **P < 0.01, ***P < 0.001, *n* = 6 each.

The experimental protocol is illustrated in Fig. 1a. Zucker fatty (ZF) and Zucker lean (ZL) rats were used to assess ORN progression. Until 32 weeks of age, ZF rats had consistently high daily food intake (Fig. 1b). Water intake and urinary volume were higher in ZF than in ZL rats until 16 weeks of age (Fig. 1c, d). At 8 weeks, ZF rats had significantly more body weight than ZL rats, which continually gained weight (Fig. 1e). The ZF group showed significantly high blood glucose levels at 16 weeks, but no significant differences were observed at other time points (Fig. 1f). Systolic blood pressure was comparable between the ZL and ZF rats (Fig. 1g). The ZF group’s heart rate increased significantly only at 16 weeks of age (Fig. 1h). At 32 weeks of age, the ZF rats had a significantly higher left kidney weight than those sacrificed (Fig. 1i). Creatinine clearance, determined using serum creatinine levels (Fig. 1j), showed an increasing trend in ZF rats compared to ZL rats (Fig. 1k), suggesting that glomerular hyperfiltration may occur in ZF rats. Furthermore, urinary protein excretion in ZF rats increased significantly after 16 weeks and remained consistent (Fig. 1l).

#### Podometric analysis

**Fig. 2:**
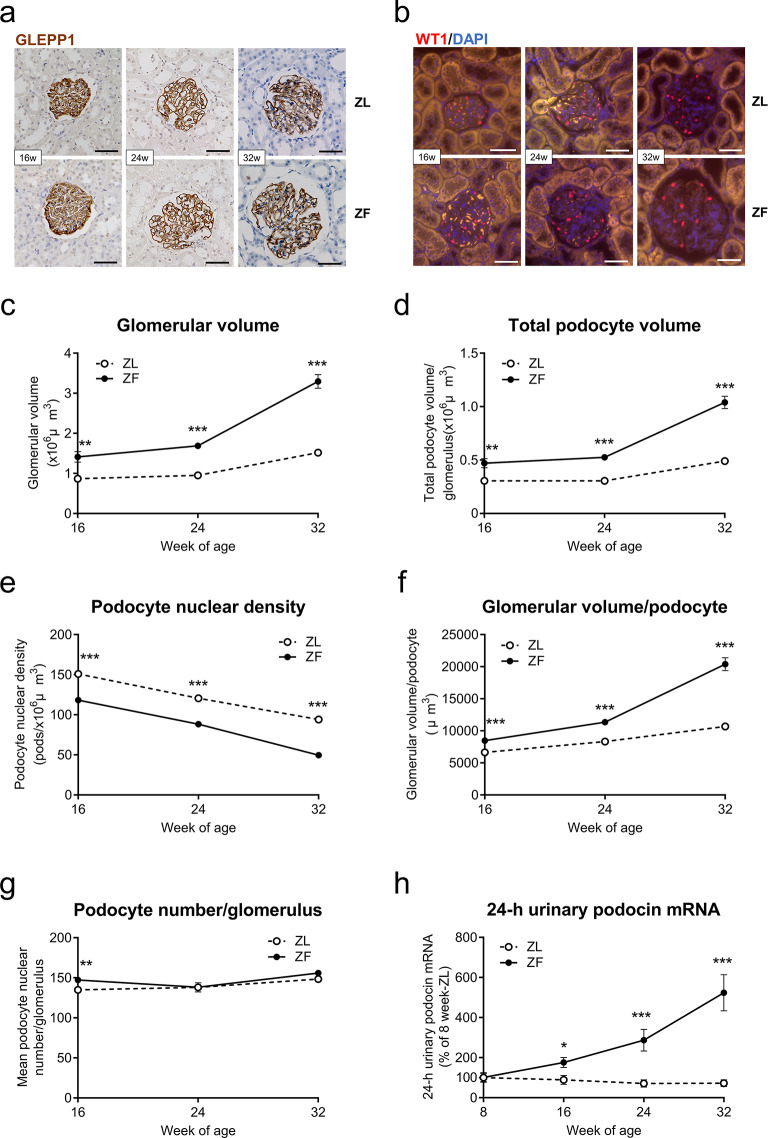
Podometric analysis in Zucker lean (ZL) and Zucker fatty (ZF) rats. **a** Representative podocytes were determined using the podocyte cytoplasmic marker glomerular epithelial protein 1 (GLEPP1) by immune-peroxidase. Scale bar = 50 μm. **b** Representative immunofluorescent results: podocytes are identified by red WT1 nuclear staining. DAPI (blue stain) shows all nuclei. Scale bar = 50 μm. **c** Glomerular volume. **d** Total podocyte volume. **e** Podocyte nuclear density. **f** Glomerular volume per podocyte. **g** Podocyte nuclear number per glomerulus. **h** 24-h urinary podocin mRNA. 24-h urinary podocin mRNA excretion is a percentage change relative to the mean excretion in 8-week-old ZL rats. Podocyte density was calculated as the number of WT1-positive nuclei normalized to glomerular volume using a stereological method as previously described by Venkatareddy et al.^67^. Glomerular and podocyte volumes in ZF rats increased while podocyte density decreased. There was no significant decrease in podocytes per glomerulus compared to ZL. A continued increase in urinary sediment podocin mRNA excretion indicates increased podocyte injury caused by podocyte hypertrophic stress in ZF rats. Data are presented as the mean ± SEM. Statistically significant differences are shown as: *P < 0.05, **P < 0.01, ***P < 0.001, *n* = 6 each.

Figure 2a–g depict representative histological outcomes and morphometry at 16, 24, and 32 weeks in ZL and ZF rats. Figure 2a and b show that the glomeruli of ZF rats were larger than those of ZL rats at all time points. The quantitative analysis indicated that the glomerular volume in ZF rats was 1.6-fold larger than that in ZL rats at 16 weeks (*P* < 0.01), which increased over time (Fig. 2c). The ZF group had 1.5 times more total podocyte volume than ZL at 16 weeks (*P* < 0.05), which continued to increase (Fig. 2d). Podocyte nuclear density (number of podocytes per glomerular volume) in ZF was consistently lower than that in ZL (*P* < 0.01; Fig. 2e). Conversely, the glomerular volume per podocyte, the reciprocal of podocyte nuclear density, increased over time in ZF rats (Fig. 2f). However, the number of podocyte nuclei per glomerulus (Fig. 2g) remained constant in both groups from 16 to 32 weeks of age, indicating that the glomerular tuft volume undertaken by a single podocyte increases in ZF rats and emphasizing the increased podocyte hypertrophic stress over time in ZF rats, with podocyte density as an important indicator of podocyte hypertrophic stress. Furthermore, podocyte hypertrophic stress can be detected noninvasively by measuring urinary sediment podocin (U-sed pod) mRNA excretion^27^. Figure 2h shows that 24-h U-sed pod mRNA excretion of ZF increased compared with ZL after 16 weeks of age, whereas levels in ZL rats remained unchanged. The findings of podocyte density and U-sed pod mRNA excretion are important indicators of podocyte hypertrophic stress^27^, and emphasize the increased podocyte hypertrophic stress over time in ZF rats.

### SGLT2i reduces podocyte injury and ORN progression

#### Time course

**Fig. 3:**
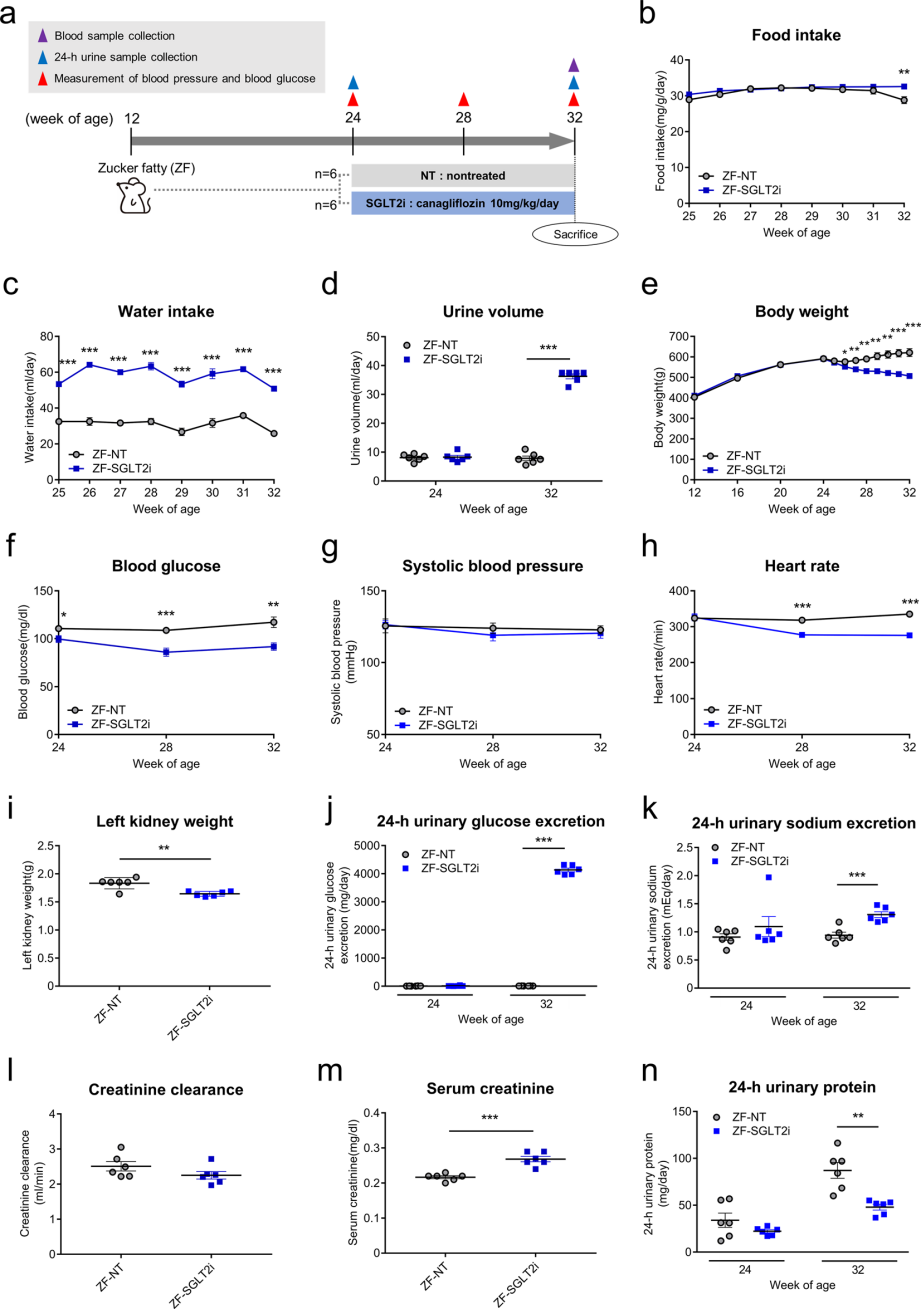
Time course of clinical parameters in nontreated (NT) Zucker fatty (ZF) rats and sodium–glucose cotransporter 2 inhibitor (SGLT2i)-treated ZF rats. **a** Schematic representation of experimental protocols. The nontreated (ZF-NT) and SGLT2i-treated (ZF-SGLT2i) groups were fed the same amount of food per body weight per day, while the ZF-SGLT2i group was treated with canagliflozin 10 mg/g/day mixed in the diet for 8 weeks beginning at 24 weeks of age. Urine samples were taken before the start of treatment and at 32 weeks of age. **b** Food intake per body weight per day. **c** 24-h water intake. **d** 24-h urine volume. **e** Body weight gain. **f** Blood glucose. **g** Systolic blood pressure. **h** Heart rate. **i** Left kidney weight at 32 weeks of age. **j** 24-h urinary glucose excretion. **k** 24-h urinary sodium excretion. **l** Creatinine clearance calculated from urinary creatinine, serum creatinine, and urine volume at 32 weeks of age. **m** Serum creatinine. **n** 24-h urinary protein. After starting SGLT2 inhibitor treatment, water intake, urinary volume, urinary glucose excretion, and urinary sodium excretion increased, while body weight, blood glucose, and urinary protein excretion decreased. The ZF-SGLT2i group’s creatinine clearance trended downward. The results of urinary sodium excretion and creatinine clearance may indicate that SGLT2 inhibitor treatment has corrected glomerular hyperfiltration. Data are presented as the mean ± SEM. Statistically significant differences are shown as: *P < 0.05, **P < 0.01, ***P < 0.001, *n* = 6 each.

We assessed the efficacy of SGLT2i in ZF rats with established ORN. As described in the protocol (Fig. 3a), nontreated (ZF-NT, *n* = 6) and canagliflozin-treated (ZF-SGLT2i, 10 mg/kg body weight/day, *n* = 6) rats were fed the same diet (mg/g body weight/day) (Fig. 3b). The ZF-SGLT2i group consumed significantly more water than the ZF-NT group (*P* < 0.001; Fig. 3c), and the ZF-SGLT2i group had 4.6 times more urinary volume after 8 weeks of treatment (*P* < 0.01; Fig. 3d). The ZF-SGLT2i group had lower body weight than the ZF-NT group by the 3rd week of treatment (*P* < 0.05), and this difference became more pronounced over time (*P* < 0.01; Fig. 3e). Blood glucose levels in the ZF-SGLT2i group were lower at 28 and 32 weeks (Fig. 3f). The two groups had similar systolic blood pressure throughout the study (Fig. 3g); however, the ZF-SGLT2i group had a significantly lower heart rate at 28 and 32 weeks (Fig. 3h). The ZF-SGLT2i group had lower left kidney weight after 32 weeks (*P* < 0.01; Fig. 3i). Additionally, the ZF-SGLT2i group showed significantly higher 24-h urinary glucose excretion (*P* < 0.01; Fig. 3j), indicating effective SGLT2i administration. The ZF-SGLT2i group also had significantly higher 24-h urinary sodium excretion (*P* < 0.01; Fig. 3k), suggesting improved sodium excretion after the SGLT2i treatment. Creatinine clearance (Fig. 3l), determined using serum creatinine (Fig. 3m), decreased in the ZF-SGLT2i group, although not significantly, suggesting a potential improvement of hyperfiltration. In addition, the ZF-SGLT2i group had significantly lower 24-h urinary protein excretion (*P* < 0.01; Fig. 3n).

#### Podometric analysis

**Fig. 4:**
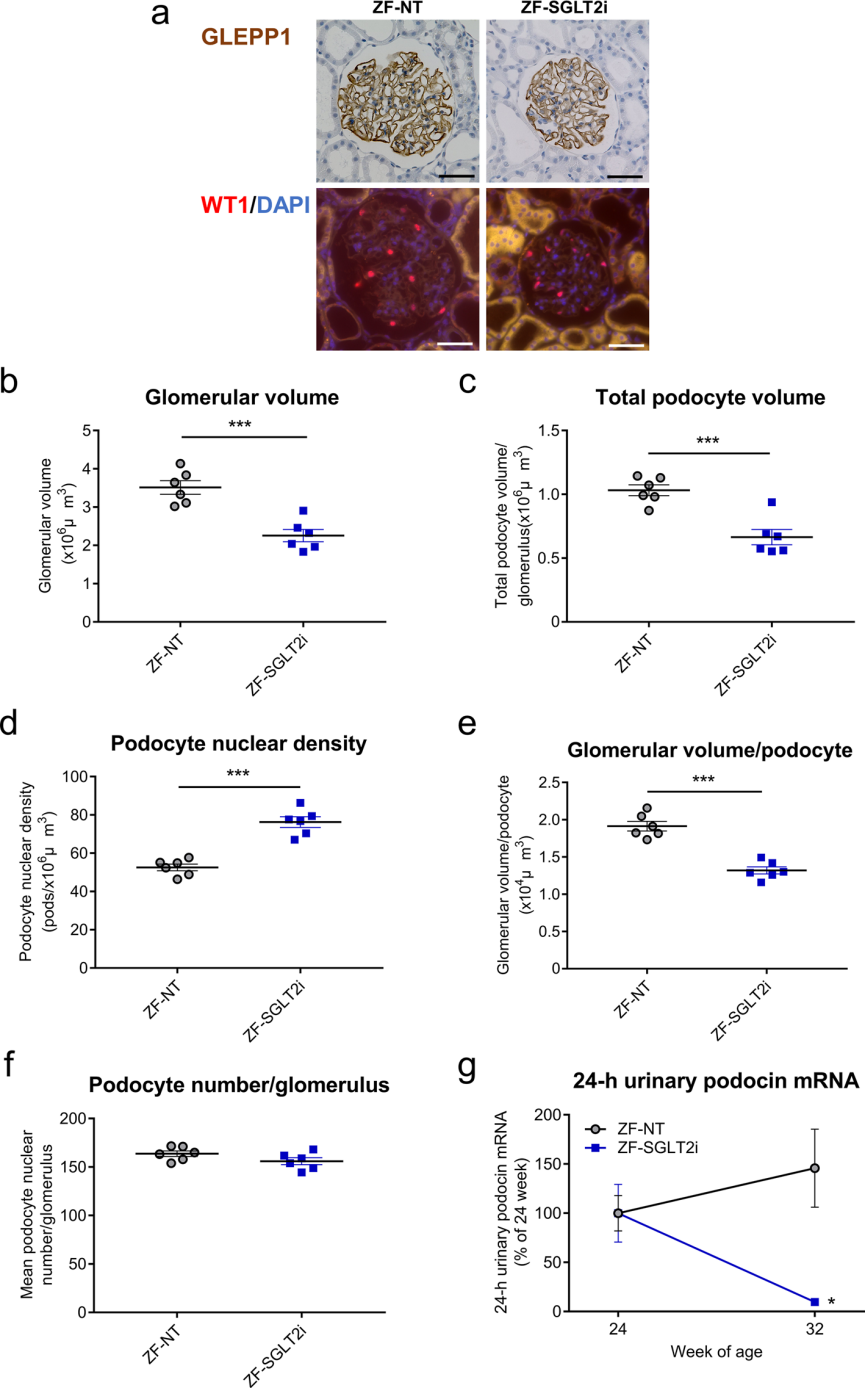
Podometric analysis in nontreated (NT) Zucker fatty (ZF) rats and sodium–glucose cotransporter 2 inhibitor (SGLT2i)-treated ZF rats at 32 weeks of age. **a** Top panels show representative podocytes identified by immunoperoxidase staining for the cytoplasmic marker glomerular epithelial protein 1 (GLEPP1). Lower panels show representative immunofluorescence results. Podocytes are identified by red WT1 nuclear staining. DAPI (blue stain) shows all nuclei. Scale bar = 50 μm. **b** Glomerular volume. **c** Total podocyte volume. **d** Podocyte nuclear density. **e** Glomerular volume per podocyte. **f** Podocyte nuclear number per glomerulus. **g** 24-h urinary podocin mRNA. 24-h urinary podocin mRNA excretion at 32 weeks of age is shown as a percentage change from the mean excretion of each group at 24 weeks of age. Podocyte density was calculated as the number of WT1-positive nuclei normalized to glomerular volume using a stereological method as previously described by Venkatareddy et al.^67^. In the ZF-SGLT2i group, glomerular and podocyte volumes were reduced while podocyte density increased. Based on histological results, urinary podocin mRNA excretion at 32 weeks in the ZF-SGLT2i group was significantly lower than at 24 weeks, indicating that SGLT2 inhibitor treatment reduces podocyte injury. Data are presented as the mean ± SEM. Statistically significant differences are shown as: *P < 0.05, ***P < 0.001), *n* = 6 each.

Figure 4a–f depict the histological and morphometric analyses at 32 weeks of ZF-NT and ZF-SGLT2i rats. Figure 4a shows that the glomeruli of ZF-SGLT2i rats were smaller than those of ZF-NT rats. Quantitative analyses showed significant reductions in glomerular and total podocyte volumes (*P* < 0.01; Fig. 4b, c). The podocyte nuclear density increased, but the glomerular volume per podocyte decreased (*P* < 0.01; Fig. 4d, e). However, the number of podocyte nuclei per glomerulus did not vary significantly between the two groups (Fig. 4f). The U-sed pod mRNA excretion of the ZF-NT group at 32 weeks of age increased compared to that at 24 weeks of age, whereas in the ZF-SGLT2i group, U-sed pod excretion at 32 weeks decreased to 9.6% of the levels at 24 weeks of age (*P* < 0.05), demonstrating that SGLT2i treatment effectively reduced podocyte injury in the ZF-SGLT2i group (Fig. 4g).

### Cyclin D1, a regulator of cell cycle progression, is associated with podocyte hypertrophy

**Fig. 5:**
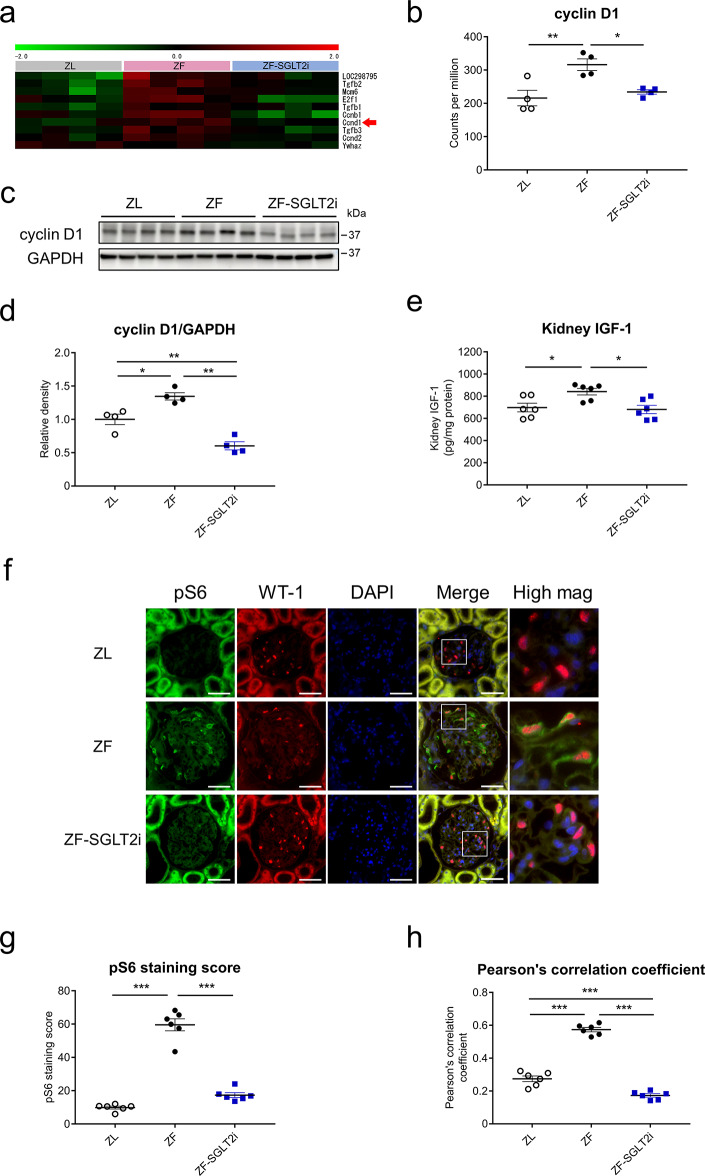
Investigation of signals underlying the development and suppression of obesity-related nephropathy revealed that the mammalian target of rapamycin (mTOR)/70 kDa ribosomal protein S6 kinase (p70S6k)/cyclin/D1 signaling pathway may be involved. **a** Total RNA was extracted from the kidney cortex collected from 32-week-old rats of Zucker lean (ZL), Zucker fatty (ZF), and sodium–glucose cotransporter 2 inhibitor (SGLT2i)-treated ZF rats. RNA sequencing (RNA-seq) was conducted, *n* = 4, each. RNA-seq detected 13,151 transcripts; 135 were related to the cell cycle. Among them, 10 genes were significantly upregulated in the nontreated ZF (ZF-NT) group compared to the ZL group (*P* < 0.05) and in ZF-NT compared to ZF-SGLT2i (*P* < 0.05). The gene heatmap depicts the relative difference from the median signal value in each gene. Black represents the median value, green represents a lower signal value, and red represents a higher signal value greater than the median. The color bar tone change represents the degree of signal value converted to log2. The red arrow points to cyclin D1. **b** A graph of gene expression signal values for cyclin D1, a key protein in the cell cycle. **c**, **d** Western blot images and quantitative analysis of cyclin D1 and glyceraldehyde-3-phosphate dehydrogenase (GAPDH) in proteins extracted from the kidney cortex at 32 weeks of age. GAPDH was used as a loading control. The original blots are presented in Supplementary Figure S1. **e** ELISA for IGF-1 in the renal cortex. **f** Representative immunofluorescence images at 32 weeks of age showing pS6 staining in the glomeruli. Single-channel (pS6, WT-1, and DAPI) and merged images are shown. Boxed regions in the merged images are presented at higher magnification in the rightmost panels (high mag). Scale bar = 50 μm. **g** Scoring phosphorylated ribosomal S6 (pS6) immunostaining in podocytes. Cyclin D1, involved in the cell cycle, was significantly higher in the ZF group in the RNA-seq results. **h** Pearson’s correlation coefficient between WT-1 and pS6 signals in glomerular tufts. These findings followed a similar pattern in the renal cortex western blots. IGF-1 and pS6 scores increased in ZF but decreased in ZF-SGLT2i. The mTOR/p70S6K/cyclin D1 signaling pathway is responsible for developing and suppressing obesity-related nephropathy. Data are presented as the mean ± SEM. Statistically significant differences are shown as: *P < 0.05, **P < 0.01, ***P < 0.001.

We propose that cell hypertrophy is vital for ORN pathogenesis. Kidney injury can result in proliferation, hypertrophy, or apoptosis, which are potentially related at the cell cycle level^32^. Proliferation necessitates normal cell cycle progression; hypertrophy occurs when cells enter the cell cycle but cannot progress past late G1 (G1/S arrest)^33^. Total RNA was isolated from the renal cortex of 32-week-old rats from three groups: ZL, ZF, and ZF-SGLT2i (*n* = 4 per group) to study the role of the cell cycle in podocyte hypertrophy and identify genes involved in ORN progression. RNA sequencing yielded 13,151 transcripts, 135 of which were identified as cell cycle-related. Genes with significant expression differences between the ZL and ZF (*P* < 0.05) and ZF and ZF-SGLT2i (*P* < 0.05) groups were identified. This approach was used to identify the 10 genes depicted in Fig. 5a. Among these, cyclin D1, a key G1 phase regulator and factor critical for cell hypertrophy and proliferation, was significantly upregulated in the ZF group compared with the ZL group. Conversely, the ZF-SGLT2i group had significantly lower cyclin D1 expression than the ZF group (*P* < 0.05; Fig. 5b–d, Supplementary Fig. S1), indicating that increased cyclin D1 expression may be related to podocyte hypertrophy.

### mTORC1 signaling pathway activation via IGF-1 modulates cyclin D1

We previously reported that growth factors activate the mTORC1 pathway, causing podocyte hypertrophic stress in a diabetic rat model^27^. The IGF-1 and mTORC1 signaling pathways regulate cyclin D1^34–37^. Similarly, IGF-1 levels in the renal cortex IGF-1 were significantly higher in the ZF group and lower in the ZF-SGLT2i group (Fig. 5e). Moreover, the phospho ribosomal S6 (pS6; a marker of mTORC1 activation) immunostaining score of podocytes was significantly higher in the ZF group than in the ZL group (*P* < 0.001) and lower in the ZF-SGLT2i group than in the ZF group (*P* < 0.001; Fig. 5f, g). In addition, colocalization analysis using Pearson’s correlation coefficient demonstrated increased association between Wilms’ Tumor 1 (WT-1) and pS6 signals within the glomerular tuft in the ZF group, which was reduced in the ZF-SGLT2i group (Fig. 5h). Overall, activating the mTOR/p70S6K/cyclin D1 signaling pathway via IGF-1 elevation plays a role in podocyte hypertrophic stress, and SGLT2i may suppress these pathways, thereby applying a protective effect on podocytes.

### SGLT2i was partially more effective than calorie restriction (CR) in preventing the progression of ORN

#### Time course

**Fig. 6:**
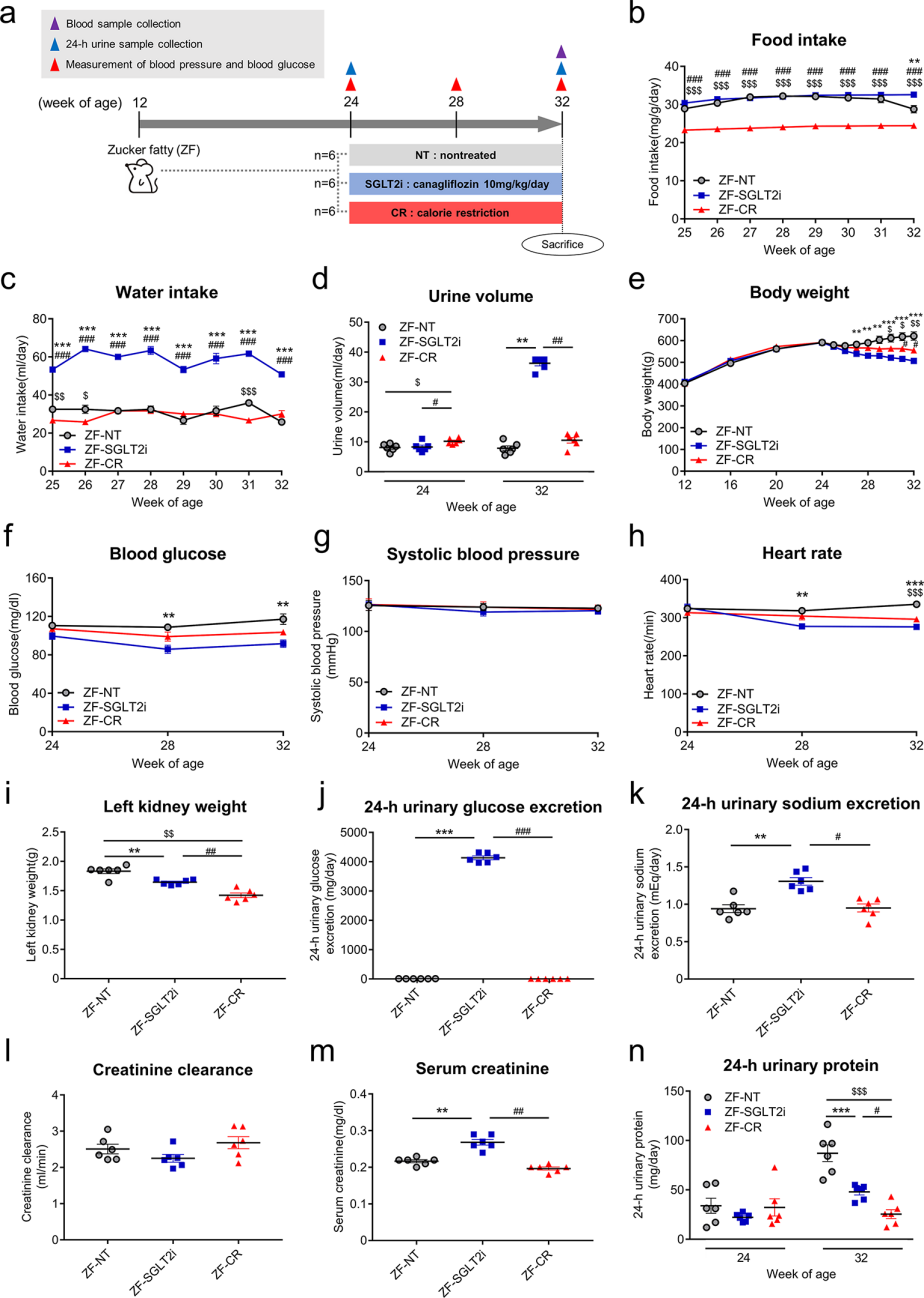
Time course of clinical parameters in nontreated (NT), sodium-glucose cotransporter 2 inhibitor (SGLT2i)-treated, and calorie restriction (CR) -treated Zucker fatty (ZF) rats. **a** Schematic representation of experimental protocols. The nontreated (ZF-NT) and SGLT2i-treated (ZF-SGLT2i) groups were fed the same amount of food per body weight daily. The ZF-SGLT2i group was treated with canagliflozin (10 mg/g/day) for 8 weeks. The ZF-CR group was placed on a 25% calorie-restricted diet to match the urinary glucose excretion of the SGLT2i group. Urine samples were collected at baseline (before SGLT2i treatment and CR) and at 32 weeks of age. **b** Food intake per body weight per day. **c** 24-h water intake. **d** 24-h urine volume. **e** Body weight gain. **f** Blood glucose. **g** Systolic blood pressure. **h** Heart rate. **i** Left kidney weight at 32 weeks of age. **j** 24-h urinary glucose excretion. **k** 24-h urinary sodium excretion. **l** Creatinine clearance was calculated from urinary creatinine, serum creatinine, and urine volume at 32 weeks of age. **m** Serum creatinine. **n** 24-h urinary protein. Body weight in the ZF-CR group decreased after the onset of CR, but to a lesser extent than in the ZF-SGLT2i group. Data are presented as mean ± SEM. Statistically significant differences are shown as: **P < 0.01, ***P < 0.001, ZF-NT versus ZF-SGLT2i, #*P* < 0.05, ##*P* < 0.01, ###*P* < 0.001, ZF-SGLT2i versus ZF-CR, $*P* < 0.05, $$*P* < 0.01, $$$*P* < 0.001, ZF-NT versus ZF-CR, *n* = 6 each.

To determine whether the improvement in podocyte hypertrophic stress and nephropathy with SGLT2i treatment was due to calorie loss or to other side effects of SGLT2 inhibition, the ZF-CR group was placed on a restricted diet. This diet reduced calorie intake by an amount equivalent to the calories lost via urinary glucose excretion in the ZF-SGLT2i group. The experimental protocol is shown in Fig. 6a. As the ZF-CR group was fed a 25% calorie-restricted diet, both the ZF-NT and ZF-SGLT2i groups consumed significantly more food throughout the study (Fig. 6b). Additionally, water intake in the ZF-SGLT2i group increased from the start of treatment (Fig. 6c), as did urinary volume at 32 weeks (Fig. 6d). Body weight was reduced in both the ZF-SGLT2i and ZF-CR groups compared to the ZF-NT group after 27 weeks of age, with the reduction being more pronounced in the ZF-SGLT2i group (Fig. 6e). The ZF-SGLT2i group had significantly lower blood glucose levels than the ZF-NT group at 28 and 32 weeks, but no group had hyperglycemia (Fig. 6f). Systolic blood pressure did not differ among the groups (Fig. 6g); however, heart rate in both the ZF-SGLT2i and ZF-CR groups were lower at 32 weeks of age (Fig. 6h). At 32 weeks of age, the left kidney of the ZF-SGLT2i group weighed more than that of the ZF-CR group and less than that of the ZF-NT group (*P* < 0.01 for both comparisons) (Fig. 6i). The ZF-SGLT2i group showed increased urinary glucose and sodium excretion at 32 weeks of age (Fig. 6j, k). Creatinine clearance (Fig. 6l) was not significantly different among the groups, but it was decreased in the ZF-SGLT2i group compared to the ZF-NT and ZF-CR groups, suggesting a correction of glomerular hyperfiltration in this group. Serum creatinine levels were higher in the ZF-SGLT2i group (Fig. 6m). Urinary protein excretion at 32 weeks was lower in both the ZF-SGLT2i and ZF-CR groups compared to the ZF-NT group, with the ZF-CR group showing a greater reduction (Fig. 6n).

#### Podometric analysis

**Fig. 7:**
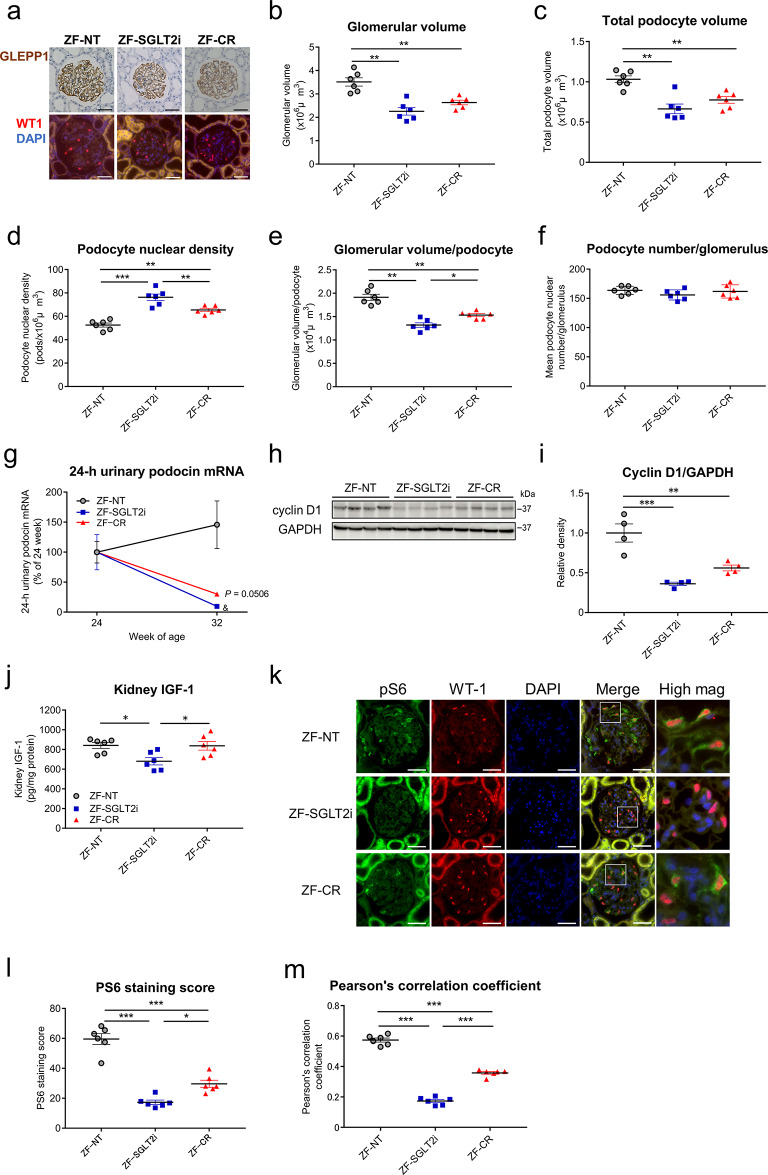
Podometric analysis and evaluation of the mammalian target of rapamycin(mTOR)/p70S6K/cyclin D1 signaling pathway in nontreated (NT), sodium-glucose cotransporter 2 inhibitor (SGLT2i)-treated, and calorie restriction (CR)-treated Zucker fatty (ZF) rats at 32 weeks. **a** Top panels show representative podocytes identified by immunoperoxidase staining for the cytoplasmic marker glomerular epithelial protein 1 (GLEPP1). Lower panels show representative immunofluorescence results. Podocytes are identified by red nuclear WT1 staining. DAPI (blue) stains all nuclei. Scale bar = 50 μm. **b** Glomerular volume. **c** Total podocyte volume. **d** Podocyte nuclear density. **e** Glomerular volume per podocyte. **f** Podocyte nuclear number per glomerulus. **g** 24-h urinary podocin mRNA. The 24-h urinary podocin mRNA excretion at 32 weeks is shown as the percentage change from the mean excretion for each group at 24 weeks. These findings suggest that SGLT2 inhibition may be more effective than CR in reducing hypertrophic stress-induced podocyte injury. **h** Representative western blots for cyclin D1 and glyceraldehyde-3-phosphate dehydrogenase (GAPDH) in the renal cortex. The original blots are presented in Supplementary Figure S2. **i** Quantitative analysis of cyclin D1 western blots. GAPDH was used as a loading control. **j** Renal cortical IGF-1 ELISA. **k** Representative immunofluorescence images at 32 weeks of age showing pS6 staining in glomeruli. Single-channel (pS6, WT-1, and DAPI) and merged are shown. Boxed regions in the merged images are presented at higher magnification in the rightmost panels (high mag). **l** Podocyte pS6 immunostaining score. **m** Pearson’s correlation coefficient between WT-1 and pS6 signals in the glomerular tufts. Renal cortical cyclin D1 expression was significantly lower in the ZF-SGLT2i and ZF-CR groups compared with the ZF-NT group. While cyclin D1 expression did not differ between the ZF-SGLT2i and ZF-CR groups, it trended lower in the ZF-SGLT2i group. The ZF-SGLT2i group had significantly lower renal cortical IGF-1 expression and podocyte pS6 staining scores than the other two groups. Data are presented as mean ± SEM. Statistically significant differences are shown as: *P < 0.05, **P < 0.01, ***P < 0.001, *n* = 4 for Western blot and *n* = 6 each for others. &*P* < 0.05, ZF-SGLT2i at 32 weeks of age versus ZF-SGLT2i at 24 weeks of age.

Figure 7a–f show the representative histological findings and morphometry at 32 weeks in the ZF-NT, ZF-SGLT2i, and ZF-CR groups. Glomerular and podocyte volumes were considerably smaller in the ZF-SGLT2i and ZF-CR groups than in the ZF-NT group. Although no significant difference was observed between the ZF-SGLT2i and ZF-CR groups, the ZF-SGLT2i group showed a reduction (Fig. 7b, c). The number of podocyte nuclei per glomerulus did not differ considerably. However, the podocyte nuclear density substantially increased and the glomerular volume per podocyte significantly decreased in the ZF-SGLT2i group (*P* < 0.01), suggesting that podocyte hypertrophic stress was more suppressed in the ZF-SGLT2i group than in the ZF-CR group (Fig. 7d–f). The rate of change in U-sed pod mRNA excretion in the ZF-CR group was insignificant, declining to 30.0% of the 24-week mean value at 32 weeks. In contrast, the ZF-SGLT2i group showed a reduction to 9.6% of the 24-week mean value (*P* < 0.05; Fig. 7g).

### SGLT2 inhibition more potently suppresses the mTOR/p70S6K/cyclin D1 signaling pathway than CR

Western blot analysis of cyclin D1 in the kidney revealed that its expression was significantly lower in the ZF-SGLT2i and ZF-CR groups than in the ZF-NT group. Although no significant difference was observed between the ZF-SGLT2i and ZF-CR groups, the ZF-SGLT2i group showed a further reduction (Fig. 7h, i, Supplementary Fig. S2). IGF-1 expression in the kidney was significantly lower in the ZF-SGLT2i group than in the ZF-NT and ZF-CR groups (Fig. 7j). Additionally, podocyte pS6 immunostaining scores were significantly lower in the ZF-SGLT2i and ZF-CR groups than in the ZF-NT group. Notably, the ZF-SGLT2i group scored significantly lower than the ZF-CR group (Fig. 7k, l). Consistent with these findings, colocalization analysis using Pearson’s correlation coefficient demonstrated reduced association between WT-1 and pS6 signals within the glomerular tuft in the ZF-SGLT2i and ZF-CR groups compared with the ZF-NT group, with a greater reduction observed in the ZF-SGLT2i group (Fig. 7m). These findings demonstrate that SGLT2 inhibition has a stronger inhibitory effect on the mTOR/p70S6K/cyclin D1 signaling pathway than CR and exerts a protective effect on podocytes by reducing hypertrophic stress.

## Discussion

In recent years, with the global increase in the prevalence of obesity, ORN has become a major public health concern. A higher body mass index is strongly linked to the onset of proteinuria, decreased estimated glomerular filtration rate (eGFR), and an increased risk of developing end-stage kidney disease^5,6^, suggesting that obesity not only constitutes a metabolic disorder but also directly affects kidney function. ORN pathogenesis involves a complex interplay of multiple pathophysiological mechanisms, including metabolic abnormalities, hemodynamic changes, inflammation, and hormonal dysregulation. Obesity leads to increased renal plasma flow and eGFR^7,8,20^, which in turn raises intraglomerular pressure, causing glomerular hypertrophy and podocyte enlargement^7,20^, contributing to kidney injury progression. Furthermore, overactivation of the RAAS has been implicated as a factor affecting kidney injury^10,11^. Endocrine abnormalities such as decreased adiponectin production and increased leptin levels^38–42^, elevated inflammatory cytokines including tumor necrosis factor-alpha and interleukin-6^14,43,44^, and ectopic lipid accumulation^17,45,46^ have been shown to contribute to kidney injury progression. ORN is a multifactorial disease and its pathogenesis involves complex interrelated mechanisms. Moreover, its underlying pathophysiology has yet to be fully elucidated. Therefore, prevention and early therapeutic intervention for ORN are critical, requiring a comprehensive management that includes obesity mitigation. Based on this background, the present study revealed two novel findings related to the pathogenesis and therapeutic targets of ORN. The first is that podocyte hypertrophic stress mediated via the mTOR/p70S6K/cyclin D1 signaling pathway plays a crucial role in ORN pathogenesis, and the second is that SGLT2i may suppress podocyte injury and nephropathy progression by inhibiting this signaling pathway.

First, we identified cyclin D1 as a candidate biomolecule that potentially contributes to ORN progression through an RNA sequencing analysis. Cyclin D1, a key regulator of cell cycle progression, is required for G1 phase progression^47^ and transition from the G0 to G1 phases^48^. Cellular hypertrophy occurs as a result of a progressive increase in cdk4/cyclin D1 kinase activity and the arrest of cells in the G1 phase of the cell cycle^33,49^. Although podocytes are terminally differentiated renal cells that cannot regenerate through proliferation, pathological stress conditions may allow podocytes to re-enter the cell cycle^50^. Cyclin D1 is abundant in cultured proliferating podocytes but not in quiescent, differentiated podocytes^51^. These findings suggest that cyclin D1 may be involved in the regulation of cell cycle-dependent processes such as hypertrophy and proliferation in podocytes.

Cyclin D1 is activated via several pathways, with the mTOR pathway being the key mediator^36^. We have reported that glomerular and podocyte hypertrophy occurs before albuminuria develops due to the activation of the IGF-1-mediated mTOR pathway, causing podocyte injury in response to hypertrophic stress in a diabetic rat model^27^. In obesity models, although mTOR pathway activation has been reported to be associated with tubulointerstitial inflammation and autophagy regulation^52,53^, evidence regarding IGF-1-dependent mTOR pathway activation in glomeruli and podocytes is currently insufficient. In this study, we demonstrated that obesity-induced elevation of IGF-1 activates the mTOR/p70S6K/cyclin D1 signaling pathway, leading to the development of glomerular hypertrophy and persistent hypertrophic stress in podocytes, which ultimately results in podocyte injury and the progression of nephropathy. These findings indicate a novel molecular mechanism underlying the development of ORN. To evaluate the mTOR signaling pathway activation in podocytes in vivo, we assessed pS6 by immunofluorescence. Given that p70S6K is a canonical substrate of mTORC1, S6 phosphorylation is widely used as a downstream readout of mTORC1 signaling in metabolic and renal studies^27,54,55^. Additionally, we quantified pS6 specifically in WT-1-positive podocytes, allowing the assessment of pathway activation at the podocyte level. Although pS6 can theoretically occur through alternative kinases, including 90 kDa ribosomal S6 kinase (p90RSK) downstream of the extracellular signal-regulated kinase pathway, under growth factor-mediated conditions, it is predominantly regulated through the mTORC1/p70S6K axis. Nevertheless, a direct measurement of phosphorylated mTOR (Ser2448) would further strengthen the evidence for mTORC1 activation. Given that phosphorylated mTOR (p-mTOR) was not examined in the present study, this represents a limitation, and future studies should include a direct assessment of p-mTOR to further confirm the activation of this pathway in ORN.

Subsequently, we investigated the effect of the SGLT2i and revealed that it exerts podocyte-protective effects by alleviating podocyte hypertrophic stress through the inhibition of mTOR/p70S6K/cyclin D1 signaling pathway. According to recent studies, the renal protective effect of SGLT2is is mediated by two primary mechanisms: (1) hemodynamic effects that restore tubuloglomerular feedback disrupted by glomerular hyperfiltration and (2) the metabolic effect that improves intracellular energy balance^31,56,57^. In this study, rats treated with an SGLT2i exhibited increased urinary sodium excretion and a tendency toward decreased creatinine clearance, suggesting that correcting glomerular hyperfiltration ameliorated glomerular hypertrophy, consistent with previous reports^58^. Notably, the present study indicated an improvement in markers of podocyte injury with SGLT2i treatment, as supported by podocyte morphological evaluation and analysis of U-sed pod mRNA, representing a mechanism distinct from the previously reported tubular-targeted effects. Furthermore, the suppression of IGF-1 and cyclin D1 expression in the renal cortex, along with reduced phosphorylation of S6 in podocytes, suggests that the SGLT2i is involved in the attenuation of podocyte hypertrophic stress via the mTOR signaling pathway. This finding has not been reported previously and constitutes the novelty of our study. The podocyte-protective effects of SGLT2is have also been reported in other pathological models. For example, in lupus nephritis, SGLT2i improves autophagy by suppressing mTORC1 activation in podocytes^59^. Similarly, in Alport syndrome, SGLT2i reduces lipotoxicity in podocytes^60^. Although these pathologies differ from ORN, these findings indicate the potential for SGLT2is to exert multifaceted protective effects on podocytes.

In this study, CR alone ameliorated podocyte/glomerular hypertrophy and suppressed mTOR activation. However, compared to CR, SGLT2i-treated rats exhibited a significant increase in podocyte density, along with reductions in U-sed pod mRNA excretion and mTOR pathway activation. This implies that the podocyte-protective effects of SGLT2i may be mediated by both CR-dependent and other mechanisms, with the molecular mechanism involving the inhibition of the IGF-1-mediated mTOR axis. Although CR can also be effective in suppressing the progression of ORN, many patients in clinical practice find it difficult to adhere to strict, long-term CR. Therefore, given the limitations of sustained dietary restriction in real-world settings, SGLT2is emerge as a practical and effective therapy for reducing podocyte injury and slowing the progression of ORN. Previous studies have shown that SGLT2 is predominantly expressed in proximal tubular epithelial cells, whereas its expression in glomerular cells, including podocytes, is extremely low^61–63^. These findings suggest that the protective effects of SGLT2is on podocytes may be mediated indirectly through systemic or intrarenal metabolic and hemodynamic mechanisms. However, in the present study, the SGLT2i group showed greater improvement in podocyte density and a greater reduction in urinary podocyte excretion compared with the CR group. These findings raise the possibility that SGLT2is may exert additional protective effects on podocytes beyond those attributable to metabolic improvement alone. SGLT2is are known to modulate intraglomerular hemodynamics by activating tubuloglomerular feedback, leading to reduced intraglomerular pressure. In the present study, detailed morphometric analyses of afferent and efferent arterioles were not performed; therefore, the contribution of glomerular hemodynamic changes could not be directly evaluated. Future studies incorporating vascular morphometric analyses will be required to clarify the relative contributions of hemodynamic and cellular mechanisms to the podocyte-protective effects of SGLT2 inhibition in ORN.

This study has some limitations. First, cyclin D1 expression was evaluated using protein extracted from the renal cortex, which contains multiple renal cell types. Therefore, the present analysis does not allow a definitive identification of the cellular source of cyclin D1 expression. Although the activation of upstream mTOR signaling was observed in WT1-positive podocytes, further studies using podocyte-specific approaches, including coimmunofluorescence staining for cyclin D1 and podocyte markers or analyses using isolated glomeruli or primary podocytes, are warranted to clarify the precise localization and functional role of cyclin D1 in podocytes. Second, direct assessment of the podocyte cell-cycle status was not performed in the present study. Given that cyclin D1 is a key regulator of cell-cycle progression, a direct evaluation of cell-cycle activity in podocytes would provide an important mechanistic insight into cyclin D1-mediated podocyte hypertrophic stress. Future studies using isolated glomeruli, primary podocytes, or dedicated cell-cycle assays will be necessary to clarify whether cyclin D1-mediated cell-cycle dysregulation contributes to podocyte injury in ORN. Third, although several previous studies have suggested the potential direct effects of SGLT2is on podocytes^30,64^, the present study did not directly evaluate SGLT2 expression in podocytes. Therefore, the precise cellular targets and mechanisms underlying the protective effects of SGLT2is on podocytes remain to be clarified. Finally, the results are based on experiments conducted in rat models, which may differ from humans, including the biological properties of podocytes and response of the mTOR pathway. In addition, although we focused on IGF-1/mTOR/cyclin D1 signaling pathway, other pathways and factors may also contribute to ORN progression, and future research is required for a comprehensive elucidation of the underlying mechanisms.

**Fig. 8:**
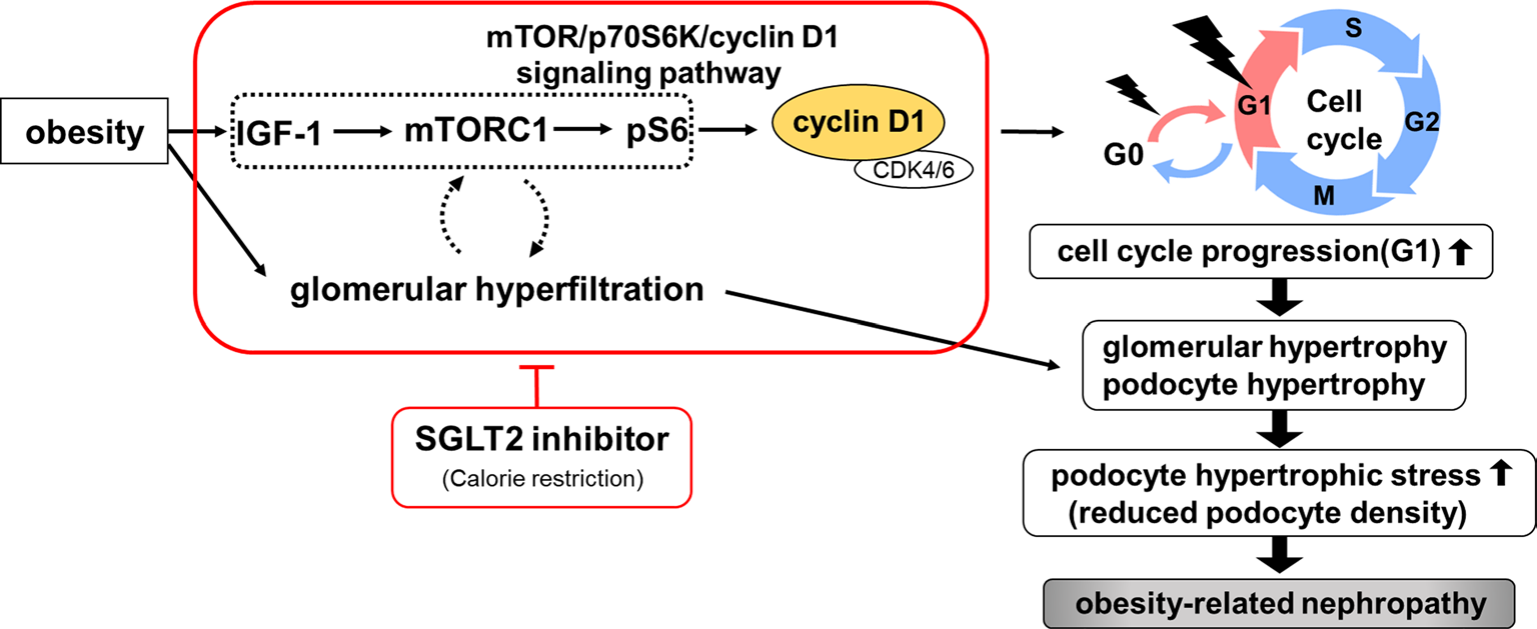
Schematic hypothesis for the progression and suppression of obesity-related nephropathy. Based on the findings of this experiment, we hypothesized that the obesity-induced increase in IGF-1 activated the mammalian target of rapamycin complex 1 (mTORC1), resulting in increased cyclin D1 expression via activation of protein S6, activating cell cycle progression and causing podocyte hypertrophy. Furthermore, glomerular hyperfiltration, caused by obesity or activating the IGF-1 and mTOR pathway, can contribute to glomerular hypertrophy and podocyte hypertrophy. The persistence of podocyte injury caused by podocyte hypertrophic stress was considered a cause of obesity-related nephropathy. These outcomes indicate that the SGLT2 inhibitor has podocyte-protective effects through calorie restriction and other effects. The molecular mechanism involves inhibiting the IGF-1-mediated mTOR/p70S6K/cyclin D1 signaling pathway.

In conclusion, this study demonstrated that obesity-induced IGF-1 elevation activates the mTOR/p70S6K/cyclin D1 signaling pathway, which promotes nephropathy progression by inducing podocyte hypertrophic stress. Furthermore, the study revealed that the SGLT2i exerts a podocyte-protective effect by inhibiting this signaling pathway, leading to the suppression of ORN progression (Fig. 8). To the best of our knowledge, this is the first report to elucidate the molecular mechanism by which SGLT2i protects podocytes by targeting the IGF-1-mediated mTOR signaling pathway. These findings provide significant evidence supporting the potential of a novel therapeutic approach for ORN.

## Methods

### Animals

Male leptin receptor-deficient ZF and ZL rats, used as obese and control models, respectively, were purchased from Japan SLC (Shizuoka, Japan). All rats were housed under controlled temperature and humidity on a 12-hour light/dark cycle. The study was approved by the Oita University Animal Research Committee (approval no. 201501 and 251501) and conducted in accordance with the approved guidelines. All methods were performed in accordance with the relevant guidelines and regulations. All animal experiments are reported in accordance with the Animal Research: Reporting of In Vivo Experiments (ARRIVE) guidelines^65^.

### ORN rat model study

Eight-week-old ZF (*n* = 6) and ZL (*n* = 6) rats underwent open kidney wedge biopsy, with approximately one-tenth of the kidney removed—sufficient to observe at least 25 glomeruli—at 16 and 24 weeks to assess pathological changes over time in an individual. The rats were anesthetized with isoflurane during kidney biopsy, and postoperative analgesia was achieved with buprenorphine. The rats were monitored until 32 weeks of age.

### SGLT2i and CR study

Eighteen ZF rats were purchased at 12 weeks of age and randomly divided into three groups (*n* = 6 per group): nontreated ZF (ZF-NT), SGLT2i-treated ZF (ZF-SGLT2i), and calorie restricted ZF (ZF-CR). Food intake (g/g body weight/day) in the ZF-SGLT2i group was adjusted to match the ZF-NT group. Beginning at 24 weeks of age, the ZF-SGLT2i group received canagliflozin (10 mg/kg/day) mixed into their food for 8 weeks. During the same period, the ZF-CR group was placed on a diet with a CR equivalent to the urinary glucose excretion observed in the ZF-SGLT2i group (an approximate 25% caloric restriction). All rats were given free water access and monitored until 32 weeks of age.

### Sample collection

Rats were anesthetized with isoflurane (3% for induction and 2% for maintenance) in oxygen. Blood samples were collected via the inferior vena cava, after which the rats were euthanized by systemic perfusion with cold physiological saline. Death was confirmed by cessation of heartbeat and respiration, following perfusion. The kidneys were then harvested and perfusion-fixed with paraformaldehyde/lysine/periodate (PLP) before being embedded in paraffin for sectioning. Blood pressure and heart rate were measured using the tail-cuff method with an automatic blood pressure monitor (BP-98 A; Softron, Tokyo). Serum creatinine, urinary protein, urinary creatinine, urinary glucose, and urinary sodium levels were calculated at a central clinical laboratory.

### RNA analysis and real-time reverse transcription-quantitative polymerase chain reaction assay of urine samples

Rats were kept in metabolic cages for 24-h urine collection to assess urine volume, proteinuria, urinary glucose, and urinary sediment podocin (U-sed pod) mRNA excretion. Urine samples were centrifuged at 3,200 ×*g* for 15 min at 4 °C; then, the supernatant was removed. The pellet was suspended in 1.5-mL diethyl pyrocarbonate-treated phosphate-buffered saline and centrifuged at 12,000 ×*g* for 5 min at 4 °C. The washed urine pellets were subsequently suspended in RLT/β-mercaptoethanol buffer (RNeasy kit; Qiagen, Germantown, MD, USA) and stored at − 80 °C. RNA was extracted from urinary pellets using an RNeasy mini kit (cat. No. 74106; Qiagen), and complementary DNAs (cDNAs) were reverse-transcribed from total RNA (~ 1 µg) using a high-capacity cDNA reverse transcription kit (Applied Biosystems, Foster City, CA, USA). Podocin mRNA abundance was quantified using a LightCycler 96^®^ system (Roche Molecular System, Mannheim, Germany) and FastStart Essential DNA Probe Master Mix (Roche Molecular System, Inc.) in a final volume of 10 µL per reaction. The TaqMan probe (Applied Biosystems) used was rat NPHS2 (podocin) (cat. No. Rn00709834_m1). cDNA standard curves were created using serially diluted standards.

### Podometric analysis

Immunofluorescence and immunoperoxidase staining employed PLP-perfused paraffin-embedded kidney sections. The mean glomerular radius (r) was measured in 25 consecutive glomerular profiles, starting from the outer cortex and progressing to the juxtamedullary glomeruli, using 2.0-µm sections. Image Pro (Media Cybernetics, Rockville, MD, USA) was used to calculate the mean glomerular radius (r), mean maximal *R* = 4/πr, and mean glomerular volume (= 4/3πR^3^)^66,67^.

The number of podocyte nuclei was determined in 25 glomerular tufts using immunofluorescence staining for WT1. Podocyte nuclear density was calculated using a stereological method as previously described by Venkatareddy et al.^67^. Following the correction for section thickness and nuclear shape, a quadratic equation was used to estimate podocyte nuclear density. The mean number of podocyte nuclei per glomerular tuft was calculated by multiplying the mean glomerular volume by the podocyte nuclear density. The glomerular epithelial protein 1 (GLEPP1)-positive tuft area was calculated from 25 consecutive glomerular tufts and expressed as the percentage of the glomerular tuft area with positive GLEPP1 immunoperoxidase staining, using Image Pro. The mean total podocyte volume was calculated by multiplying the mean glomerular volume by the mean percentage of GLEPP1-positive areas. Reagents: Primary antibody was WT1 (SC-7385 monoclonal IgG1; Santa Cruz Biotechnology, USA) at 1:50, with a Cy3-labeled secondary antibody (Jackson ImmunoResearch Laboratories, USA) at 1:100. Slides were coverslipped using SlowFade Gold antifade reagent with DAPI (S36939; Invitrogen, USA). Mouse monoclonal anti-GLEPP1 antibody (1B4) was used at 1:100 (a gift from Dr. Wiggins’ laboratory) with the Vectastain Elite ABC immunoperoxidase kit (PK-6100; Vector Laboratories Inc, USA). All morphometric analyses were performed by investigators blinded to the group allocation.

### Scoring of phospho S6 immunofluorescence staining

Phosphorylation of ribosomal protein S6 (pS6) was evaluated as a downstream indicator of mTORC1/p70S6K signaling activity. The intensity of pS6 staining in podocytes was quantified across 25 glomeruli. Podocytes were identified by nuclear staining for WT-1. The intensity of pS6 staining in podocytes within the glomerular tuft was measured using ImageJ (NIH, USA) and scored on a scale of 0 to 255 (representing unstained areas and maximum intensity, respectively). Colocalization between the WT-1 and p-S6 signals was analyzed using the Coloc2 plugin in ImageJ. Pearson’s correlation coefficients were calculated within the glomerular tuft. For each rat, 25 glomeruli were analyzed and the mean value per rat was used for statistical comparisons. A primary polyclonal phospho-S6 antibody (#2211; Cell Signaling Technology, USA) was used at a 1:400 dilution, followed by a FITC-labeled secondary antibody (1:100; Jackson ImmunoResearch Laboratories, USA). Slides were mounted using SlowFade Gold Antifade Mountant with DAPI (S36939; Invitrogen, USA).

### Renal cortex IGF-1

The rats were euthanized, and the renal cortex was harvested and stored at − 80 °C until use. A 100 mg sample of renal cortex was homogenized in 600 µL of phosphate-buffered saline and diluted tenfold prior to the assay. Renal cortical IGF-1 levels were measured using a Rat IGF1 ELISA kit (Cat. No. MBS824704; MyBioSource.com, USA).

### RNA sequencing

Total RNA was extracted from renal tissue samples collected at 32 weeks of age using an RNeasy kit (Qiagen, Germantown, MD, USA). Samples with an RNA Integrity Number (RIN) > 7 and a total of 1 µg of RNA were used as input for sequencing. Sequencing libraries were prepared using the MGIEasy rRNA Depletion Kit and the MGIEasy RNA Directional Library Prep Set. The resulting libraries were quality-controlled and sequenced on the MGI DNBSEQ G400 FAST platform to generate 150 bp paired-end reads.

### Western blotting

Total protein was extracted from renal cortices by homogenization in RIPA buffer. Protein samples were separated by SDS-PAGE, transferred to PVDF membranes (Bio-Rad Laboratories, Inc., Hercules, CA), and blocked with 5% non-fat milk in Tris-buffered saline containing 0.01% Tween-20. The membranes were then washed and incubated overnight at 4 °C with anti-cyclin D1 (1:1,000, Proteintech Group, Inc.). Following subsequent washes, the membranes were incubated with peroxidase-conjugated secondary antibodies and visualized using an ECL chemiluminescence kit (Cytiva, Tokyo, Japan). To determine equal loading, the membranes were stained with anti-glyceraldehyde-3-phosphate dehydrogenase (1:20,000, Sigma-Aldrich). The autoradiographs were scanned with ImageQuant 800 (Cytiva, Tokyo, Japan).

### Statistical methods

Data are presented as mean ± SEM. Statistical analyses were performed using GraphPad Prism version 7. Comparisons between two groups were performed using an unpaired two-tailed Student’s t-test or a paired t-test, as appropriate. For comparisons among three groups, statistical significance was determined by one-way analysis of variance followed by Tukey’s *post hoc* test. A *P*-value of < 0.05 was considered statistically significant.

## Supplementary Information

Below is the link to the electronic supplementary material.

Supplementary Material 1

## Data Availability

The RNA-seq data have been deposited in the Gene Expression Omnibus database accession number GSE285091 (https://www.ncbi.nlm.nih.gov/geo/query/acc.cgi? acc=GSE285091). Additional data supporting the findings of this study are available from the corresponding author upon request.
