# Supplementary material for: Sodium–glucose cotransporter 2 inhibitor ameliorates podocyte hypertrophic stress by suppressing the mTOR/p70S6K/cyclin D1 signaling pathway in obesity-related nephropathy
Source: Sci Rep. 2026 Apr 20;16:18385. doi: 10.1038/s41598-026-49025-w (PMC13266057; doi:10.1038/s41598-026-49025-w)
Supplement: Supplementary file 1 — Supplementary Material 1 [file 41598_2026_49025_MOESM1_ESM.pdf]

Sodium–glucose cotransporter 2 inhibitor ameliorates podocyte hypertrophic stress by suppressing the mTOR/p70S6K/cyclin D1 signaling pathway in obesity-related nephropathy

Miho Suzuki[1], Akihiro Fukuda[1], Ryo Kurimoto[1], Akiko Kudo[1], Hirotaka Shibata[1]

[1]Department of Endocrinology, Metabolism, Rheumatology and Nephrology, Faculty of Medicine, Oita University, Yufu, Japan

(a) cyclin D1

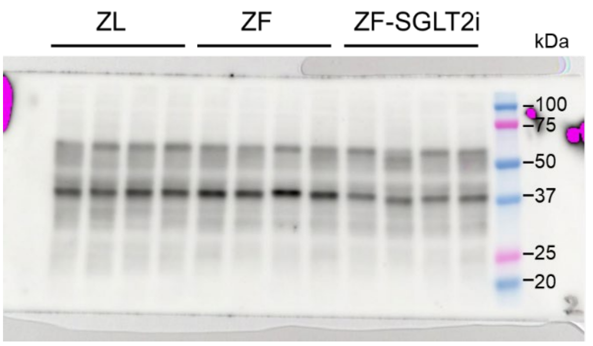

(b) GAPDH

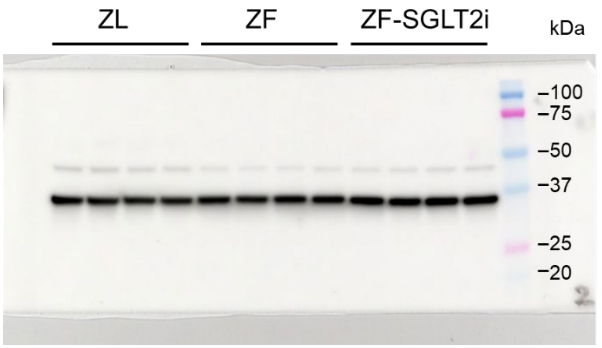

Supplementary Figure S1. Full-length western blot images of cyclin D1 and glyceraldehyde-3-phosphate dehydrogenase (GAPDH) in proteins extracted from the kidney cortex of 32-week-old Zucker lean (ZL), Zucker fatty (ZF), and SGLT2 inhibitor (SGLT2i)-treated ZF rats. (a)Cyclin D1. (b)GAPDH. GAPDH was used as a loading control.

(a) cyclin D1

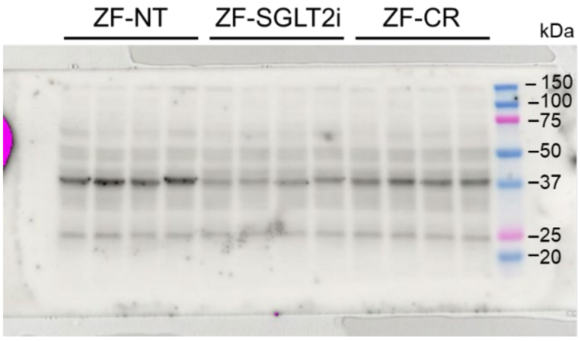

(b) GAPDH

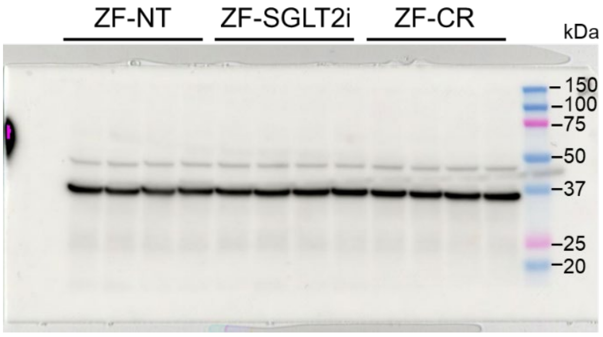

Supplementary Figure S2. Full-length western blot images of cyclin D1 and glyceraldehyde-3-phosphate dehydrogenase (GAPDH) in proteins extracted from the kidney cortex of 32-week-old nontreated (NT), sodium-glucose cotransporter 2 inhibitor (SGLT2i)-treated, and calorie restriction (CR)-treated Zucker fatty (ZF) rats. (a)Cyclin D1. (b)GAPDH. GAPDH was used as a loading control.
